# Evaluation of FFR and CPET in patients with *de novo* coronary chronic total occlusion treated with DCB vs. DES

**DOI:** 10.3389/fcvm.2025.1584548

**Published:** 2025-07-31

**Authors:** Lingli Guo, Hengdao Liu, Xi Zhao, Ting Liu, Chenyang Gao, Jihao Duan, Dongfeng Wang, Junwei Zhao, Yuzhen Wei, Ruipeng Song, Hailong Tao

**Affiliations:** ^1^ Gastroscopy Center, The Third People’s Hospital of Henan Province, Zhengzhou, Henan, China; ^2^ Department of Cardiology, The First Affiliated Hospital of Zhengzhou University, Zhengzhou, Henan, China; ^3^ Department of Cardiology, Neihuang People’s Hospital, Anyang, Henan, China; ^4^ Department of Cardiology, Luohe Third People’s Hospital, Luohe, Henan, China; ^5^ Department of Cardiology, Yexian People’s Hospital, Pingdingshan, Henan, China

**Keywords:** coronary chronic total occlusion, drug-coated balloon, fractional flow reserve, cardiopulmonary exercise testing, drug-eluting stent

## Abstract

**Background:**

Drug-coated balloon (DCB) is a novel therapeutic strategy for *de novo* coronary chronic total occlusion (CTO). However, fractional flow reserve (FFR), a key indicator of evaluating coronary function, and cardiopulmonary exercise testing (CPET), an important indicator of cardiopulmonary function, are rarely reported for evaluating the effectiveness of DCB in CTO lesions.

**Methods and results:**

In this retrospective study, 100 patients were enrolled and classified into the DCB group (*n* = 48) and the drug-eluting stent (DES) group (*n* = 52). All patients underwent coronary angiography immediately after PCI and during follow-up. Some patients underwent FFR measurement (*n* = 64) or CPET (*n* = 56) at follow-up. There was no significant difference in baseline clinical characteristics between the two groups. Compared with the DES group, the DCB group had a significantly smaller late lumen loss (LLL) (*P* < 0.05). Also, there was no significant difference in the proportion of FFR values ≥0.90 between the two groups at follow-up. Similarly, there was also no statistically significant difference in the CPET parameters between the two groups (*P* > 0.05). In addition, the incidence of MACE (major adverse cardiovascular events) showed no statistical difference during hospitalization and follow-up between the two groups (*P* > 0.05).

**Conclusions:**

DCB treatment for *de novo* CTO lesions yields FFR and CPET results comparable to DES, with an even smaller LLL. This result provides a new approach for the treatment of *de novo* CTO lesions.

## 1 Introduction

*De novo* coronary chronic total occlusion (CTO) refers to the complete obstruction of one or more coronary arteries for more than 3 months, which is the last bastion of interventional therapy for coronary artery disease (CAD). The cornerstone of treating CTO lesions lies in effective revascularization, which has the potential to markedly lower the occurrence of major adverse cardiovascular events (MACE). Percutaneous coronary intervention (PCI), a minimally invasive revascularization procedure, has demonstrated encouraging outcomes in lowering cardiac death and improving postoperative patient outcomes (1, 2). Presently, drug-eluting stent (DES) implantation is recognized as an important PCI technique for treating CTO lesions. However, the insertion of a metallic stent may result in various immediate and long-term complications related to the stent. Moreover, some researchers found that the inherent properties of CTO lesions made it challenging to choose a stent with a precise diameter for PCI therapy (3, 4). Furthermore, the duration of dual antiplatelet therapy (DAPT) may last for a year or more after PCI with DES (5).

As a maturing therapeutic approach, drug-coated balloon (DCB) has demonstrated effective clinical results for both *de novo* small-vessel disease and high bleeding risk (6). Also, it has become the treatment of choice for in-stent restenosis (7). Growing evidence suggests that DCB exhibits more advantages in vascular reconstruction compared with DES, avoiding permanent metallic implants, reducing DAPT duration (6 vs. 12 months), and promoting vessel remodeling. Recently, research has shown that treating *de novo* CTO solely with DCB leads to a smaller late lumen loss (LLL) and acceptable MACE rates compared with DES implantation (8). Actually, diverse measurements are available to obtain incremental information, although coronary angiography (CAG) is the conventional technique for directing PCI and evaluating outcomes after PCI (9). Fractional flow reserve (FFR) and cardiopulmonary exercise testing (CPET), the innovation evaluation indicators of this study, are worth mentioning. FFR, regarded as the “gold standard” for diagnosing moderate stenosis in patients suffering from coronary heart disease, is capable of evaluating the functional significance of coronary stenosis (10). CPET provides a reproducible quantification of cardiopulmonary function and prognostic stratification in chronic coronary syndromes. It also served as an efficacious testing procedure in evaluating the efficacy of CTO PCI in a recent study (11, 12).

In a word, FFR and CPET testing after DCB treatment for CTO disease are extremely essential for evaluating coronary function and cardiopulmonary function. Consequently, with the DES group serving as a control group, we sought to delve deeper into the efficacy and safety of DCB in the treatment of CTO lesions through flow reserve fraction measurement and cardiopulmonary exercise testing.

## 2 Methods

### 2.1 Study population

A total of 996 consecutive patients underwent CTO PCI across our center at the First Affiliated Hospital of Zhengzhou University from March 2018 to January 2021. In this retrospective observational study, 100 patients, diagnosed with *de novo* CTO and underwent PCI, were enrolled in this study. Of these, 64 patients (64%) were prospectively enrolled and completed follow-up with FFR assessment at 3–12 months post-PCI. Based on treatment strategies, these patients were divided into the DCB group (*n* = 48) and the DES group (*n* = 52). The inclusion criteria were as follows: (1) 18–80 years of age; (2) meeting the diagnostic criteria of *de novo* CTO, an occlusion with the absence of antegrade flow through the lesion with a presumed or documented duration of ≥3 months (13); (3) repeat coronary angiography in our hospital with ≥3 months of follow-up. The exclusion criteria were as follows: (1) acute coronary artery occlusion and in-stent restenosis; (2) severely impaired hepatic and renal function and malignant tumors; (3) severe valvular disease and chronic obstructive pulmonary disease; (4) severe allergy to anticoagulants and contrast agents; (5) incomplete data. All subjects gave their informed consent to participate in this study. This study was approved by the Ethics Committee of the First Affiliated Hospital of Zhengzhou University (2022-KY-0799).

### 2.2 PCI procedure

Obstructive CAD was defined as at least one coronary artery vessel stenosis of ≥75%, and PCI was recommended. Preoperatively, all patients received DAPT before and after PCI, including aspirin (Bayer, Germany, loading dose 300 mg) + clopidogrel (Sanofi, France, loading dose: 300 mg) or aspirin 300 mg + ticagrelor (AstraZeneca, UK, loading dose: 180 mg). Patients received maintenance treatment (maintenance dose: aspirin 100 mg day^−1^ + clopidogrel 75 mg day^−1^, or aspirin 100 mg day^−1^ + ticagrelor 90 mg, twice daily). The choice of the interventional strategy (DCB or DES strategy) was left to the discretion of the experienced. On the one hand, we determined the type of PCI and the method of opening CTO lesions based on the latest version of the “Recommended Approach for Interventional Treatment of Chronic Total Occlusion Coronary Arteries in China,” with contemporary drug-coated balloon and contemporary drug-eluting stents that have obtained CE certification and FDA approval as unified consumables. On the other hand, DCB angioplasty was executed solely when the residual stenosis was ≤30%, with either no dissection or only type A or B dissection after predilation balloon angioplasty; otherwise, it was recommended to apply DES implantation without a DCB.

With regard to DCB, according to quantitative coronary angiography (QCA) results, a size-matched DCB was chosen, with a balloon-to-vessel ratio of 0.8–1.0 and an inflation pressure of 8–10 atm (1 atm = 101.325 kPa), maintained for approximately 60 s. The criteria for successful treatment were residual stenosis ≤30% at the target lesions with Thrombolysis in Myocardial Infarction (TIMI) grade III flow, and without type C or higher dissection, failing which salvage DES implantation would be executed. With regard to DES, in the process of DES implantation, after predilation, DES with a stent-to-vessel ratio of 1.1–1.2 was selected according to the vessel condition, and the length exceeded the target lesion by 3–5 mm. The DES was delivered to the target lesion and released subsequently. To ensure that the stent was fully dilated against the wall, sufficient postdilatation was carried out using a non-compliant balloon.

In general, after the PCI procedure, patients in the DCB group receive DAPT regimen for at least 6 months and those in the salvage DES implantation group as well as the DES group receive it for at least 12 months. The use of additional medications depends on the supervising physicians.

### 2.3 Follow-up observation

#### 2.3.1 Quantitative coronary angiography

Immediately after PCI and between 3 and 12 months after the procedure, every patient underwent CAG measurement. The CAG data of all patients were analyzed by using QCA software, including (1) immediate postoperative reference vessel diameter (RVD), minimum lumen diameter (MLD), and degree of stenosis (DS) [DS = (1 − MLD/RVD) × 100%]; (2) MLD, DS, LLL (MLD after intervention minus MLD at follow-up): it refers to the reduction in the minimal lumen diameter of a coronary artery between the immediate postintervention period. Late lumen enlargement (LLE, follow-up MLD minus post-PCI MLD): it describes an increase in lumen diameter at follow-up compared with the postprocedural measurement.

#### 2.3.2 Flow reserve and cardiopulmonary exercise test

FFR measurement was performed in selected patients including the DCB group (*n* = 30) and the DES group (*n* = 34) at 3–12 months’ follow-up, adhering to a standard FFR measurement procedure. All patients who underwent FFR measurement did not have restenosis.

During a follow-up period of 3–12 months, CPET was conducted on chosen patients, notably those in the DCB group (*n* = 28) and DES group (*n* = 28), and the standardized procedure was carried out as per standard procedures (14). The main parameters included peak oxygen uptake (peakVO_2_), anaerobic threshold (AT), oxygen pulse (VO_2_ pulse), and work efficiency slope (ΔVO_2_/ΔWR).

FFR and CPET were performed based on patient consent, clinical stability, and the absence of contraindications. Not all patients underwent these tests due to logistical constraints (e.g., patient refusal, lack of equipment availability).

#### 2.3.3 Postoperative complications

At 3–12 months’ follow-up, we observed the occurrence of MACE, including cardiac death, non-fatal myocardial infarction (MI), and restenosis (15).

### 2.4 Statistical analysis

SPSS version 27.0 (SPSS, Chicago, IL, USA) was used for statistical analysis. Categorical data were expressed as frequencies and analyzed by using chi-square or Fisher's exact test. Quantitative data were expressed as mean ± standard deviation (SD) or median (interquartile range). Normally distributed data were analyzed by using a Student’s *t*-test, and non-normally distributed data were analyzed by using a Wilcoxon rank-sum test. A Kaplan–Meier curve was used to describe survival analysis. A two-tailed *P* < 0.05 was considered statistically significant.

## 3 Results

### 3.1 Baseline demographic and clinical characteristics

There was no statistically significant difference in age, gender, creatinine levels, and left ventricular ejection fraction (LVEF) (*P* > 0.05). Risk factors linked to coronary heart disease, such as hypertension, hyperlipidemia, diabetes, smoking, and familial coronary artery disease history, showed no significant differences between the two groups (*P* > 0.05). Furthermore, there was no statistically significant differences in the history of PCI, stable angina, unstable angina Syntax score, and the Japanese chronic total occlusion (J-CTO) score between the two groups (*P* > 0.05) (Table 1).

**Table 1:** Baseline patient characteristics of the study population.

Variable	DCB (*n* = 48)	DES (*n* = 52)	*P*-value
Age (years)	57.93 ± 10.35	58.94 ± 09.47	0.491
Males, *n* (%)	34 (70.83)	32 (61.5)	0.327
With hypertension, *n* (%)	25 (52.08)	24 (46.2)	0.553
With hyperlipidemia, *n* (%)	15 (31.25)	15 (28.8)	0.793
With diabetes, *n* (%)	13 (27.08)	10 (19.2)	0.351
Smoking history, *n* (%)	17 (35.42)	14 (26.9)	0.359
PCI history, *n* (%)	6 (12.50)	4 (7.7)	0.423
Family history of CHD, *n* (%)	14 (29.17)	16 (30.8)	0.861
Creatinine (μmol L^−1^)	76.44 ± 21.19	73.35 ± 19.46	0.52
LVEF (%)	61.23 ± 5.60	60.51 ± 7.10	0.70
Stable angina, *n* (%)	17 (35.42)	15 (28.8)	0.482
Unstable angina, *n* (%)	28 (58.33)	26 (50.0)	0.404
J-CTO score	2.1 ± 0.8	2.3 ± 0.7	0.38
Syntax score	15.2 ± 5.1	16.0 ± 4.8	0.45

DCB, drug-coated balloon; DES, drug-eluting stent; PCI, percutaneous coronary intervention; CHD, coronary heart disease; LVEF, left ventricular ejection fraction; J-CTO score, the Japanese chronic total occlusion score.

Values are mean ± standard deviation or number (%).

### 3.2 QCA analysis

The median follow-up time was 8 months for the DCB group and 10 months for the DES group. The postoperative and follow-up results of CAG of all patients are given in Table 2. The DCB group exhibited a significantly smaller MLD and a greater DS than the DES group (*P* < 0.05) immediately after PCI. However, at follow-up, there was no statistically significant difference in MLD and DS between the two groups (*P* > 0.05). In addition, the value of LLL observed in the DCB group was significantly smaller than that in the DES group at follow-up (*P* < 0.01).

**Table 2:** Results of QCA in the immediate postoperative period and at follow-up between the DCB and the DES groups.

Variable	DCB (*n* = 48)	DES (*n* = 52)	*P*-value
Postprocedure			
RVD (mm)	2.26 ± 0.59	2.62 ± 0.56	0.236
MLD (mm)	1.67 ± 0.59	2.17 ± 0.38	<0.01
DS (mm)	26.59 ± 16.53	20.75 ± 12.46	0.048
Follow-up			
RVD (mm)	2.26 ± 0.59	2.62 ± 0.56	0.236
MLD (mm)	1.70 ± 0.72	1.97 ± 0.80	0.07
DS, M(P25, P75) (%)	22.80 (7.97, 36.68)	24.59 (10.98, 38.91)	0.47
LLE, *n* (%)	32 (66.76)	30 (57.7)	0.356
LLL, M (P25, P75) (mm)	−0.18 (−0.29, 0.27)	0.18 (−0.38, 0.59)	<0.01

QCA, quantitative coronary angiography; DCB, drug-coated balloon; DES, drug-eluting stent; RVD, reference vessel diameter; MLD, minimal lumen diameter; DS, diameter stenosis; LLE, late lumen enlargement; LLL, late lumen loss.

Values are mean ± standard deviation or number (%).

### 3.3 FFR and CPET analysis

At follow-up, merely a small number of patients underwent FFR measurements, including the DCB group (*n* = 30) and the DES group (*n* = 34). At follow-up, the FFR showed no statistically significant difference between the two groups (0.9021 ± 0.0175 vs. 0.9053 ± 0.0180). Similarly, there was no significant difference in the percentage of patients with FFR ≥ 0.9 between the two groups (Table 3). In addition, 28 patients from both the DCB and the DES groups underwent cardiopulmonary exercise testing at follow-up. The main CPET parameters, including peakVO_2_, AT, VO_2_ pulse, and ΔVO_2_/ΔWR, were analyzed, and none of the differences between the two groups were statistically significant (*P* > 0.05) (Table 4).

**Table 3:** Results of FFR parameters in the DCB and DES groups at follow-up.

Variable	DCB (*n* = 34)	DES (*n* = 30)	*P*-value
FFR	0.9021 ± 0.0175	0.9053 ± 0.0180	0.464
FFR ≥ 0.90, *n* (%)	21 (61.76)	21 (70)	0.489
FFR ≤ 0.80, *n* (%)	0 (0)	0 (0)	—

FFR, fractional flow reserve; DCB, drug-coated balloon; DES, drug-eluting stent.

Values are mean ± standard deviation or number (%).

**Table 4:** Results of CPET parameters in the DCB and DES groups at follow-up.

Variable	DCB (*n* = 28)	DES (*n* = 28)	*P*-value
peakVO_2_ (mL min^−1^)	1,611.14 ± 169.61	1,618.79 ± 166.02	0.896
VO_2_ pulse peak (mL beat^−1^)	13.65 ± 1.93	13.42 ± 2.02	0.660
ΔVO_2_·ΔWR^−1^ [(mL/min) W^−1^]	11.69 ± 2.25	11.42 ± 2.22	0.658
AT (mL kg^−1^ min^−1^)	39.27 ± 4.66	39.64 ± 4.10	0.752

CPET, cardiopulmonary exercise testing; DCB, drug-coated balloon; DES, drug-eluting stent; peakVO_2_, peak oxygen uptake; VO_2_ pulse, oxygen pulse; ΔVO_2_·ΔWR^−1^, work efficiency slope; AT, anaerobic threshold.

Values are mean ± standard deviation or number (%).

### 3.4 Safety analysis

Table 5 summarizes the postoperative complications. In the DCB group, one patient (2.1%) underwent salvage stenting implantation for intraprocedural dissection. During angiographic follow-up, restenosis was observed in five patients (9.6%) of the DES group, of which two patients (3.8%) were reoccluded, and in the DCB group, restenosis occurred in seven target vessels (14.58%), of which one target vessel (2.1%) was completely reoccluded with TIMI flow grade 0. There was no significant difference in the incidence of restenosis between the two groups (*P* = 0.649). No in-hospital and follow-up complications, such as cardiac death and non-fatal MI, were observed in this study. For the overall incidence of MACE during in-hospital and follow-up, there was no significant difference between the DCB group [14.58% (7/48)] and the DES group [9.62% (5/52)] (*P* = 0.454). Although not statistically significant, the numerically higher restenosis rate in the DCB group (14.58% vs. 9.62%) suggests a potential signal that warrants further investigation in larger trials.

**Table 5:** Adverse events at follow-up in patients with *de novo* coronary CTO between the DCB and the DES groups.

Variable	DCB (*n* = 48)	DES (*n* = 52)	*P*-value
Salvage stenting implantation, *n* (%)	1 (2.1)	0 (0.0)	—
The total of MACE, *n* (%)	7 (14.58)	5 (9.6)	0.649
Cardiac death, *n* (%)	0 (0.0)	0 (0.0)	—
Non-fatal MI, *n* (%)	0 (0.0)	0 (0.0)	—
Restenosis, *n* (%)	7 (14.58)	5 (9.6)	0.649
Reoccluded, *n* (%)	1 (2.1)	2 (3.8)	0.602

CTO, chronic total occlusion; DCB, drug-coated balloon; DES, drug-eluting stent; MACE, major adverse cardiovascular events, MI, myocardial infarction.

Values are mean ± standard deviation or number (%).

**Figure 1:**
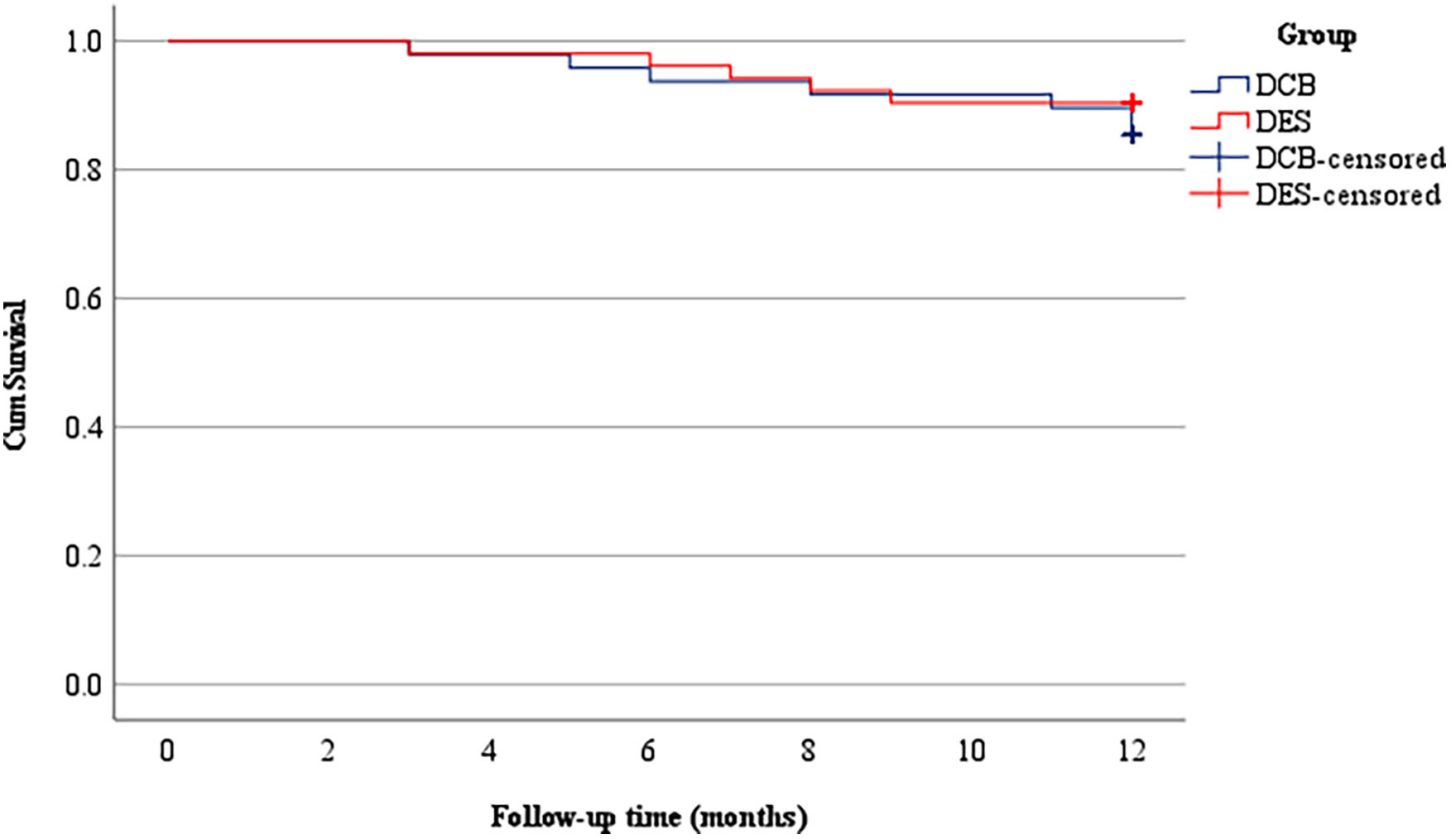
The survival analysis curve of occurrence of MACE in two groups after PCI. Log-rank, *P* = 0.464. MACE, major adverse cardiovascular events; PCI, percutaneous coronary; DCB, drug-coated balloon; DES, drug-eluting stent; PCI, percutaneous coronary intervention; CHD, coronary heart disease; LVEF, left ventricular ejection fraction. Values are mean ± standard deviation or number (%).

The Kaplan–Meier survival analysis showed no significant difference between the two groups at a 12-month follow-up (Log-rank, *P* = 0.464). For each patient, the survival event is the occurrence of MACE, and the censoring status is the end of follow-up or loss to follow-up (Figure 1).

## 4 Discussion

This research verifies that solely using DCB for CTO is an optional, relatively effective, and secure method for coronary intervention, with less LLL, similar FFR values, and CPET parameter values compared with DES implantation.

In this study, we found a smaller MLD and higher DS in the DCB group immediately after PCI, in contrast to the DES group (*P* < 0.05). This could be attributed to the vessel's sudden elastic recoil following the immediate aftermath of balloon withdrawal, the balloon's relatively conservative inflation pressure, and the lack of postoperative stenting for luminal expansion and support. Consistent with a prior study (8), LLE was more frequent in the DCB group (*P* = 0.356), although not statistically significant. The smaller LLL in DCB (*P* < 0.01) reflects positive remodeling, likely due to the absence of metallic scaffolding. Several factors can contribute to these findings. Initially, without metal remnants after DCB treatment, the damaged vessels are prone to remodel aggressively, leading to a steady enlargement of the vessel diameter. In addition, the less late lumen loss is also related to the healing of dissection flaps, regression of plaque or dissection flap, preprocedure thick-cap fibro of the Roman plaques, and postoperative deep dissection reaching the tunics media (16, 17).

We found no statistically significant difference in the flow reserve fraction between the DCB and the DES groups [(0.9021 ± 0.0175) vs. (0.9053 ± 0.0180)] during the follow-up period. However, it may be limited by incomplete follow-up data (only 64% had FFR measurements), risking selection bias. The numerically higher restenosis rate in DCB (14.58% vs. 9.62%) could skew FFR results if excluded cases differed clinically. The small sample size and partial testing reduce statistical power to detect true differences. Larger studies with mandatory FFR assessment and balanced restenosis analysis are needed to confirm equivalence. Research indicates that an FFR ≥ 0.90 after percutaneous coronary intervention correlates with a lower likelihood of major adverse cardiovascular events (18). Our data revealed that at follow-up, the occurrence rates of FFR ≥ 0.90 in the DCB group and DES group were [61.76% (21/34)] vs. [70% (21/30)], respectively (*P* > 0.05). Despite a higher proportion of FFR ≥ 0.90 in the DES group compared with the DCB group, there was no statistically significant difference between them. We did not perform FFR measurements in patients with restenosis. Consequently, no FFR values ≤0.80 were recorded in either group during follow-up. Nonetheless, earlier studies indicate that FFR below the clinical threshold for revascularization (FFR ≤ 0.80) occurs in <1% to 36.5% of patients after PCI (19–21). The absence of such values in our cohort may reflect this exclusion criterion and the limited FFR sample size.

Cardiopulmonary exercise testing offers a comprehensive assessment of cardiac function and exercise capacity. The assessment of VO_2_/WR, VO_2_ pulse, and ventilatory efficiency of CPET have been shown to be more sensitive and specific in identifying induced myocardial ischemia and perfusion defects than just exercise ECG alone (11, 22, 23). Moreover, cardiopulmonary exercise testing parameters can be used as symptom-related outcome indicators for evaluating the benefits after CTO PCI. It has been shown that in addition to relieving local ischemia and improving exercise capacity after CTO PCI, it is also associated with an increase in peakVO_2_ and in AT (1, 11, 12). Consequently, in our research, we selected specific CPET parameters, including peakVO_2_, AT, as well as VO_2_ pulse and ΔVO_2_/ΔWR, which can reflect the degree of ischemia. At follow-up, CPET parameters were comparable between groups, with no statistically significant differences (*P* > 0.05), which suggests that the CPET outcomes in the DCB group were comparable to those in the DES group.

The safety analysis revealed a reduced restenosis rate in the DES group (9.6%) compared with the DCB group (14.58%), but the difference was not statistically significant. In 2013, the PEPCAD-CTO trial pioneered the exploration of DCB in treating CTO lesions, evaluating the effect of DCB combined with bare-metal stents (BMSs). Also this research found that the restenosis rate of DCB combined with BMS was 27.7% (24). In our research, the restenosis rate was significantly less than the PEPCAD-CTO study, which may be related to improved DCB techniques, including high-pressure predilation and prolonged balloon inflation, which likely contributed to lower restenosis rates. Subsequently, Koln et al. (25) observed an 11.8% rate of restenosis in CTO lesions solely treated with DCB, which is similar to our observations. A comparable incidence of MACE was observed at a 12-month postoperative follow-up, with no significant difference between the DCB group [14.58% (7/48)] and the DES group [9.62% (5/52)] (*P* = 0.649). The survival analysis showed no significant difference between the two groups during the follow-up period. Although MACE rates showed no statistically significant difference (14.58% vs. 9.62%, *P* = 0.454), the numerically higher rate in the DCB group may suggest a clinically relevant signal. This study was underpowered to confirm safety equivalence; larger trials are needed to determine whether this trend reflects a true difference.

### 4.1 Study limitation

This study has some limitations. First, it is a single-center, retrospective, non-randomized observational study with a small sample size, increasing susceptibility to selection bias and unmeasured confounders. Second, only 64% of patients underwent FFR measurements, and 56% completed CPET, further reducing statistical power and potentially skewing results toward healthier patients (e.g., due to financial constraints or poor adherence). Third, FFR was not assessed in cases of restenosis, which may have masked true differences in functional outcomes between groups. Last but not least, the numerically higher restenosis rate in the DCB group (14.58% vs. 9.62%) warrants caution in interpreting safety equivalence. Therefore, multicenter, randomized controlled clinical trials with larger samples are needed to further investigate the use of DCB or DES after CTO revascularization compared with cardiopulmonary exercise testing.

## 5 Conclusions

1. DCB achieved comparable functional outcomes to DES, with no significant differences in FFR or CPET parameters.
2. DCB was associated with significantly less LLL and greater LLE than DES, indicating favorable vessel remodeling
3. A numerically higher restenosis rate was observed with DCB (14.58% vs. 9.62%), although it was statistically insignificant; this trend merits investigation in larger trials.

These findings support DCB as a viable strategy for *de novo* CTO lesions, but further validation is needed.

## Data Availability

The raw data supporting the conclusions of this article will be made available by the authors, without undue reservation.
